# Noticing education campaigns or public health messages about vaping among youth in the United States, Canada and England from 2018 to 2022

**DOI:** 10.1093/her/cyad044

**Published:** 2024-01-02

**Authors:** Katherine East, Eve Taylor, Erikas Simonavičius, Matilda Nottage, Jessica L Reid, Robin Burkhalter, Leonie Brose, Olivia A Wackowski, Alex C Liber, Ann McNeill, David Hammond

**Affiliations:** National Addiction Centre, Institute of Psychiatry, Psychology and Neuroscience, King’s College London, Addictions Sciences Building, 4 Windsor Walk, London SE5 8BB, UK; School of Public Health Sciences, University of Waterloo, 200 University Avenue West, Waterloo, Ontario N2L 3G1, Canada; National Addiction Centre, Institute of Psychiatry, Psychology and Neuroscience, King’s College London, Addictions Sciences Building, 4 Windsor Walk, London SE5 8BB, UK; National Addiction Centre, Institute of Psychiatry, Psychology and Neuroscience, King’s College London, Addictions Sciences Building, 4 Windsor Walk, London SE5 8BB, UK; National Addiction Centre, Institute of Psychiatry, Psychology and Neuroscience, King’s College London, Addictions Sciences Building, 4 Windsor Walk, London SE5 8BB, UK; School of Public Health Sciences, University of Waterloo, 200 University Avenue West, Waterloo, Ontario N2L 3G1, Canada; School of Public Health Sciences, University of Waterloo, 200 University Avenue West, Waterloo, Ontario N2L 3G1, Canada; National Addiction Centre, Institute of Psychiatry, Psychology and Neuroscience, King’s College London, Addictions Sciences Building, 4 Windsor Walk, London SE5 8BB, UK; School of Public Health, Rutgers, The State University of New Jersey, 683 Hoes Lane West, Piscataway, NJ 08854, USA; Cancer Prevention and Control, Lombardi Comprehensive Cancer Center, Georgetown University, 3800 Reservoir Road NW, Washington, DC 20007, USA; National Addiction Centre, Institute of Psychiatry, Psychology and Neuroscience, King’s College London, Addictions Sciences Building, 4 Windsor Walk, London SE5 8BB, UK; School of Public Health Sciences, University of Waterloo, 200 University Avenue West, Waterloo, Ontario N2L 3G1, Canada

## Abstract

Public health campaigns have the potential to correct vaping misperceptions. However, campaigns highlighting vaping harms to youth may increase misperceptions that vaping is equally/more harmful than smoking. Vaping campaigns have been implemented in the United States and Canada since 2018 and in England since 2017 but with differing focus: youth vaping prevention (United States/Canada) and smoking cessation (England). We therefore examined country differences and trends in noticing vaping campaigns among youth and, using 2022 data only, perceived valence of campaigns and associations with harm perceptions. Seven repeated cross-sectional surveys of 16–19 year-olds in United States, Canada and England (2018–2022, *n* = 92 339). Over half of youth reported noticing vaping campaigns, and noticing increased from August 2018 to February 2020 (United States: 55.2% to 74.6%, AOR = 1.21, 95% CI = 1.18-1.24; Canada: 52.6% to 64.5%, AOR = 1.13, 1.11-1.16; England: 48.0% to 53.0%, AOR = 1.05, 1.02-1.08) before decreasing (Canada) or plateauing (England/United States) to August 2022. Increases were most pronounced in the United States, then Canada. Noticing was most common on websites/social media, school and television/radio. In 2022 only, most campaigns were perceived to negatively portray vaping and this was associated with accurately perceiving vaping as less harmful than smoking among youth who exclusively vaped (AOR = 1.46, 1.09-1.97). Consistent with implementation of youth vaping prevention campaigns in the United States and Canada, most youth reported noticing vaping campaigns/messages, and most were perceived to negatively portray vaping.

## Introduction

There is strong scientific consensus internationally that vaping e-cigarettes is less harmful than smoking cigarettes, but is not risk-free and should be discouraged among youth and people who have never smoked [1–3]. Vaping can help people to quit smoking [4] and nicotine-containing e-cigarettes are recommended in some countries, such as the UK and New Zealand, for adults to quit or reduce their smoking [5]. Despite this, there are pervasive misperceptions that vaping is equally or more harmful than smoking [1, 6]; for example, among youth, only 38% in the United States, 45% in Canada and 63% in England accurately perceived that vaping is less harmful than smoking in 2020, a reduction from 61%, 66% and 77%, respectively, in 2017 [6]. Similar trends have been seen among adults [1]; for example, among adults who smoke in Great Britain, the proportion who held this accurate perception has declined from 60% in 2014 to 34% in 2023 [7].

Education campaigns (strategic, active efforts to educate the public) or public health messages (any public health statements or messages) have provided information about vaping in several countries, including absolute harms (i.e. compared to not vaping) and harms relative to smoking cigarettes [8–18]. However, the content of campaigns and messages, as well as the target audience, differs across countries. In the United States and Canada, vaping campaigns and associated messages from public health organisations have focused predominantly on youth vaping prevention [8–15, 18]. For example, in the United States in 2018, national campaigns aiming to prevent vaping among youth were launched (e.g. ‘The Real Cost’ in September 2018, and the ‘Truth’ campaign which ran from October to December 2018) [8–11] as well as several state and regional campaigns [14, 15, 18]. Similarly, in Canada, a national campaign aiming to prevent youth from vaping was launched in December 2018, followed by the national ‘Consider the Consequences of Vaping’ campaign in February 2019 [12], as well as provincial youth vaping prevention campaigns over the same period [13]. In England, since 2017, vaping has featured only in national campaigns aiming to help adults quit smoking [19, 20], and, since 2015, there have been regional vaping campaigns [21] and widely publicized annual reports [1] containing messages that vaping is less harmful than smoking but is not risk-free, and that people who have never smoked should not take up vaping.

Expenditures on youth vaping prevention campaigns in the United States and Canada have also been greater than expenditures on any vaping campaigns in England [11, 12, 22]. For example, in the United States, ‘The Real Cost’ has been described as ‘a nearly $60 million effort’ [23] and the advocacy organisation Campaign for Tobacco-Free Kids partnered with Bloomberg Philanthropies in 2019 to launch a US$160 million campaign entitled ‘Protect Kids: Fight Flavored E-Cigarettes’ [11]. In Canada, CDN$9 million was invested into Health Canada’s ‘Consider the Consequences of Vaping’ campaign [12]. In England, there has been no specific government expenditure on vaping campaigns, other than the aforementioned smoking cessation campaigns which mention vaping [22].

Understanding the extent to which the public are exposed to vaping campaigns or messages is important because campaigns/messages can change public perceptions of vaping harms [1, 24, 25] particularly when provided by public health bodies which are viewed as credible sources of information [25–27]. Evidence suggests that vaping campaigns/messages aiming to deter youth vaping and highlighting that vaping is harmful can increase perceptions that vaping is harmful [1, 25]; however, they can also result in overestimation of the harms of vaping relative to smoking [1, 25]. Conversely, campaigns/messages highlighting that vaping is less harmful than smoking can increase the accurate perception that vaping is less harmful than smoking [1, 25, 28], but have also been found to increase misperceptions that vaping is safe [28]. It is possible that youth vaping prevention campaigns in the United States and Canada have contributed to the increasing misperception that vaping is equally or more harmful than smoking observed among youth in these countries [6]. It is also possible that vaping campaigns or messages could help to correct misperceptions [1, 24, 25]. Ideally, messages would convey that vaping is risky, but less so than smoking, in line with current evidence [1–3].

Specific channels may allow for targeted interventions communicating vaping information to either youth or adults who smoke. National campaigns in the United States [8–11] and Canada [12] have attempted to deter youth from vaping through disseminating information via television, online videos, social media, dedicated websites and schools; for example, in the United States, school youth vaping prevention campaigns have been found to increase perceptions of vaping as harmful among school children [29, 30]. Conversely, pharmacies/chemists may be ideal settings for communicating the benefits of switching to vaping to adults who smoke [31], particularly because evidence suggests that pharmacy staff are often asked by people who smoke for evidence-based advice around vaping [32] and pharmacies can help to increase the efficacy of smoking cessation interventions [33]. Bars or other adult-only venues could also be used to target vaping information to adults. However, little is known about youth exposure to vaping campaigns or messages across these, or other, channels.

This study therefore aimed to examine country differences (England, Canada, United States; Aim 1) and trends (from 2018 to 2022; Aim 2) in the prevalence of noticing vaping campaigns or messages among youth, overall and via individual channels. We had two a-priori hypotheses [34]: first, that noticing any vaping campaigns or messages would be more prevalent among youth in the United States and Canada compared with England, because vaping campaigns in the United States and Canada predominantly targeted youth and received higher expenditure than those in England; second, that noticing any vaping campaigns or messages would increase between 2018 and February/March 2020 among youth in the United States, Canada and England, because campaigns were launched in all three countries over this period [8–10, 12–15, 20, 21]. Moreover, the 2019 outbreak of lung injuries associated with vaping contaminated cannabis products in the United States led some public health organizations (e.g. the United States Centres for Disease Control and Prevention) to warn against the use of e-cigarettes, and also increased public discussions about the health harms of vaping internationally; exposure to public health messaging about vaping may therefore have also increased in late 2019 and early 2020. The second hypothesis is restricted to survey waves up to February/March 2020 because the COVID-19 pandemic impacted vaping behaviours [35], dominated public health messaging, and disrupted education. Using the most recent wave of data (2022) only, we also explored the perceived valence of the vaping campaigns or messages that youth noticed and associations between noticing negative campaigns and vaping harm perceptions (Aim 3).

## Methods

The analysis plan for this study was pre-registered, and code made available, on the Open Science Framework (osf.io/6c2uz) [34].

### Data source

Data were from seven waves (2018 to 2022) of the International Tobacco Control Policy Evaluation Project (ITC) Youth Tobacco and Vaping Survey, a repeat cross-sectional online survey of youth aged 16–19 in England, Canada and the United States. Samples were recruited from the Nielsen Consumer Insights Global Panel and their partners’ panels. Respondents were recruited either directly or through their parents via email invitations sent to panelists (after targeting for age criteria) including those known to be parents. The surveys were online and took approximately 20 minutes to complete. This study received ethics clearance through the University of Waterloo Research Ethics Committee (ORE#21847/31017) and the King’s College London Psychiatry, Nursing & Midwifery Research Ethics Subcommittee. A full description of the study methods can be found in the Technical Reports [36]. The 2018 survey wave was selected as the first wave for analyses because this was the first in which respondents asked about their noticing of education campaigns or public health messages about vaping.

A total of *n* = 99 977 respondents completed the surveys, of whom *n* = 92 339 were retained in the analytic sample. Respondents were excluded if they: failed data integrity checks (*n* = 3450), had missing/incomplete data on variables required for calculating weights or determining smoking or vaping status (*n* = 1862), were recruited in a previous wave (*n* = 2220; to maintain repeat cross-sectional data, as some cohort respondents were present in the first few waves), or were an ineligible age (*n* = 106).

### Measures

#### Noticing education campaigns or public health messages about vaping

All respondents were asked, ‘In the past 12 months, have you noticed education campaigns or public health messages about e-cigarettes / vaping in any of the following places? …’ followed by a list of channels (shown in Table I), with response options ‘Yes,’ ‘No,’ ‘Don’t know’ or ‘Refuse to answer’ for each. Respondents who answered ‘Yes’ to any of the channels were coded as having noticed any vaping campaigns or messages in the past 12 months; all other respondents were coded as ‘Other’. Each individual channel was also modelled as an outcome where the proportion who responded ‘Yes’ to noticing via that channel was at least 5% of the overall sample. All channels (1 to 17 listed above) were noticed by at least 5% of the overall sample and so were modelled as outcomes, except ‘other (please specify)’, which was reported by <1% of the sample.

**Table I.** Differences between England, Canada, and the United States in the proportion of youth who reported noticing vaping campaigns or public health messages overall and via individual channels (n = 92 339)

		England as reference		Canada as reference	
	%	AOR (95% CI)	*P*	AOR (95% CI)	*P*
Any noticing					
England	52.5	**REF**			
Canada	65.3	**1.62 (1.56-1.68)**	**<0.001**	**REF**	
United States	72.1	**2.34 (2.24-2.44)**	**<0.001**	**1.45 (1.39-1.51)**	**<0.001**
On websites or social media, like Facebook, Twitter, YouTube, Instagram or Snapchat					
England	26.9	**REF**			
Canada	39.3	**1.68 (1.61-1.75)**	**<0.001**	**REF**	
United States	48.3	**2.57 (2.46-2.68)**	**<0.001**	**1.53 (1.47-1.59)**	**<0.001**
At school					
England	23.3	**REF**			
Canada	41.9	**2.32 (2.23-2.42)**	**<0.001**	REF	
United States	42.0	**2.40 (2.30-2.51)**	**<0.001**	1.03 (0.99-1.07)	0.099
On television or radio					
England	17.4	**REF**			
Canada	28.4	**1.84 (1.76-1.93)**	**<0.001**	**REF**	
United States	42.7	**3.56 (3.40-3.73)**	**<0.001**	**1.93 (1.86-2.01)**	**<0.001**
On billboards or posters					
England	14.5	**REF**			
Canada	21.5	**1.54 (1.47-1.62)**	**<0.001**	**REF**	
United States	29.4	**2.44 (2.33-2.57)**	**<0.001**	**1.58 (1.51-1.65)**	**<0.001**
At a chemist/pharmacy					
England	18.5	**REF**			
Canada	23.6	**1.29 (1.24-1.35)**	**<0.001**	REF	
United States	22.8	**1.29 (1.22-1.35)**	**<0.001**	0.99 (0.95-1.04)	0.774
In shops/stores that sell e-cigarettes/vaping products					
England	17.1	REF			
Canada	17.5	0.98 (0.94-1.03)	0.446	REF	
United States	18.8	**1.10 (1.04-1.15)**	**<0.001**	**1.12 (1.06-1.17)**	**<0.001**
In print newspapers or magazines					
England	14.2	**REF**			
Canada	16.3	**1.10 (1.05-1.16)**	**<0.001**	**REF**	
United States	19.4	**1.42 (1.35-1.50)**	**<0.001**	**1.29 (1.23-1.35)**	**<0.001**
Outside shops/stores that sell e-cigarettes/vaping products					
England	15.3	**REF**			
Canada	16.7	1.04 (0.99-1.10)	0.105	**REF**	
United States	18.4	**1.21 (1.15-1.28)**	**<0.001**	**1.17 (1.11-1.23)**	**<0.001**
In leaflets/flyers					
England	15.0	**REF**			
Canada	13.4	**0.81 (0.76-0.85)**	**<0.001**	**REF**	
United States	17.7	**1.19 (1.13-1.25)**	**<0.001**	**1.48 (1.40-1.55)**	**<0.001**
Taxis or buses/public transit					
England	11.4	**REF**			
Canada	17.0	**1.46 (1.38-1.54)**	**<0.001**	**REF**	
United States	14.2	**1.25 (1.18-1.33)**	**<0.001**	**0.86 (0.81-0.90)**	**<0.001**
At kiosks or temporary sales locations (in shopping centres, parked in the street, other places, but not at specific events)					
England	11.9	**REF**			
Canada	11.5	**0.90 (0.85-0.95)**	**<0.001**	**REF**	
United States	12.5	1.03 (0.97-1.09)	0.331	**1.15 (1.08-1.22)**	**<0.001**
At events like fairs, markets, festivals, sporting events or music concerts					
England	8.5	**REF**			
Canada	10.7	**1.19 (1.12-1.27)**	**<0.001**	**REF**	
United States	12.6	**1.52 (1.43-1.62)**	**<0.001**	**1.28 (1.20-1.35)**	**<0.001**
At the cinema/movies					
England	6.7	**REF**			
Canada	10.4	**1.48 (1.38-1.58)**	**<0.001**	**REF**	
United States	13.7	**2.17 (2.02-2.32)**	**<0.001**	**1.47 (1.38-1.56)**	**<0.001**
In email or text messages					
England	6.4	**REF**			
Canada	9.1	**1.36 (1.27-1.46)**	**<0.001**	**REF**	
United States	12.6	**2.07 (1.93-2.22)**	**<0.001**	**1.51 (1.42-1.61)**	**<0.001**
At work					
England	7.2	**REF**			
Canada	9.3	**1.22 (1.14-1.31)**	**<0.001**	**REF**	
United States	9.6	**1.35 (1.26-1.45)**	**<0.001**	**1.10 (1.03-1.18)**	**0.003**
In bars or pubs					
England	8.5	**REF**			
Canada	8.0	**0.87 (0.82-0.94)**	**<0.001**	**REF**	
United States	8.3	0.95 (0.88-1.02)	0.127	**1.08 (1.01-1.16)**	**0.029**
In regular postal mail					
England	6.0	**REF**			
Canada	6.9	1.07 (1.00-1.16)	0.066	**REF**	
United States	8.8	**1.47 (1.37-1.59)**	**<0.001**	**1.37 (1.28-1.48)**	**<0.001**

All data except *n* are weighted, and all data are aggregated across all seven survey waves (2018–2022).

Estimates are from separate logistic regression models (one per outcome) adjusting for demographic covariates (age group, sex, race/ethnicity, perceived family financial situation, student status) and survey wave; see Table S2 for the full models.

‘Other’ was not modelled as an outcome because it was reported by <1% of the sample.

a Wording differed according to country: At a [chemist (UK)/ pharmacy (CA, US)]; In [UK = leaflets, CA, US = flyers]; At the [UK = cinema/CA-US = movies].

Bolded values are where *P*<.05.

#### Country and survey wave (independent variables)

##### Country.

England, Canada, United States.

##### Survey wave.

August/September 2018, August/September 2019, February/March 2020, August 2020, February/March 2021, August 2021, August/September 2022. Survey wave was treated as categorical to aid interpretation of the findings. Inclusion of the August 2021 and August/September 2022 survey waves was additional to the pre-registration [34] due to the availability of data from these waves at the time of analysis and because a new measure examining perceived valence of vaping campaigns was added in August/September 2022.

### Covariates

#### Age group.

16–17, 18–19.

#### Sex.

Male, female. Sex was coded from sex at birth for most respondents, or imputed from gender where sex at birth was missing [36].

#### Race/ethnicity.

White only, any other race/ethnicity, don’t know/refused. Race/ethnicity was derived from country-specific items that are described in the Technical Reports [36].

#### Perceived family financial situation.

Not meeting basic expenses, just meeting basic expenses, meeting needs with a little left over, living comfortably, don’t know/refused.

#### Student status.

Yes (enrolled currently or for upcoming year), no, don’t know/refused.

#### Smoking/vaping subgroups

We considered five mutually exclusive use subgroups as a sensitivity analysis, with categories based on prior research [37–40]: 1) exclusive past 30-day vaping (i.e. vaped but did not smoke in the past 30 days); (2) exclusive past 30-day smoking (i.e. smoked but did not vape in the past 30 days); (3) past 30-day vaping/smoking (i.e. vaped and smoked in the past 30 days); (4) ever but not past 30-day vaping/smoking (i.e. ever smoked and/or vaped, but not in the past 30 days); (5) never vaped/smoked (never smoked and never vaped).

#### Perceived valence of vaping campaigns and vaping harm perceptions in 2022

##### Perceived valence of vaping campaigns.

Additional to the pre-registration [34], in the most recent (August/September 2022) wave only, a new measure was added examining perceived valence of vaping campaigns. Respondents who selected ‘Yes’ for any of the above channels for noticing vaping campaigns were subsequently asked, ‘Were the majority of education campaigns or public health messages you noticed about e-cigarettes…’ (a) ‘Mostly negative about e-cigarettes,’ (b) ‘Mostly positive about e-cigarettes,’ (c) ‘About the same number of positive and negative,’ (d) ‘Don’t know’, or (e) ‘Refuse to answer’. Responses were coded as mostly negative (a) versus otherwise (b-e).

##### Vaping harm perceptions.

Additional to the pre-registration [34], for this study, we examined harm perceptions of vaping relative to smoking in the most recent (August/September 2022) wave only, using the measure ‘Is using e-cigarettes/vaping less harmful, about the same, or more harmful than smoking cigarettes?’ with response options (a) ‘A lot more harmful than “regular” tobacco cigarettes,’ (b) ‘A little more harmful than “regular” tobacco cigarettes,’ (c) ‘As harmful as “regular” tobacco cigarettes’, (d) ‘A little less harmful than “regular” tobacco cigarettes’, (e) ‘A lot less harmful than “regular” tobacco cigarettes’, (f) ‘Don’t know’, and (g) ‘Refused to answer’. Responses were coded as accurately perceiving vaping as less harmful than smoking (d-e) versus otherwise (a-c or f-g), consistent with prior research [6, 21, 31].

### Analyses

To address Aims 1 and 2, the number and proportion of youth who noticed any vaping campaigns or public health messages, as well as via each individual channel, were reported by country and survey wave. Logistic regression models adjusting for survey wave and demographic covariates were used to predict noticing any vaping campaigns or messages, as well as each individual channel, from country. A country-by-survey wave interaction term was subsequently added to each of the adjusted logistic regression models, and outcomes were contrasted between survey waves within countries using Stata’s *margins* post-estimation command. To address our hypotheses, England and August/September 2018 were primarily treated as reference categories for country and survey wave, respectively. These analyses were conducted with the overall sample, as well as sensitivity analyses stratified by each vaping/smoking subgroup.

To address Aim 3, using data from August/September 2022 only, the number and proportion of youth who perceived that campaigns or messages were mostly positive or mostly negative towards vaping were reported by country, and logistic regression models adjusting for demographic covariates were used to predict the perception that campaigns were mostly negative (versus other) from country. Also using data from August/September 2022 only, logistic regression models adjusting for country and demographic covariates were used to predict accurate (versus other) relative harm perceptions from the perception that campaigns were mostly negative (versus other) towards vaping. The interaction between perceiving campaigns to be negative and country were explored.

Analyses were conducted in Stata v.17 and applied cross-sectional post-stratification sample weights (see Technical Reports for details) [36].

## Results

### Sample characteristics

Table S1 (Supporting Information) shows the sample characteristics by wave and country. Most participants identified as White (only) (66.5%), were students (91.9%), and perceived their family’s financial situation as meeting needs or living comfortably (68.8%). The majority reported that they had never smoked or vaped (53.4%), while 9.5% reported only vaping in the past 30 days, 4.8% reported only smoking in the past 30 days, 5.4% reported both smoking and vaping in the past 30 days, and 26.7% reported ever vaping and/or smoking but not in the past 30 days.

### Noticing education campaigns or public health messages about vaping

**Fig. 1.**
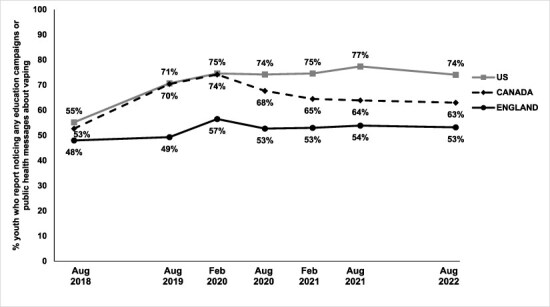
Proportion of youth aged 16–19 who reported noticing any education campaigns or public health messages about vaping within England, Canada and the United States, 2018 to 2022 (*n* = 92 339). Data are weighted.

Over most years, more than half of youth in England, Canada, and the United States reported noticing vaping campaigns or public health messages (Fig. 1).

#### Country differences (Aim 1)

Table I shows the country differences in noticing vaping campaigns or messages, overall and via 17 individual channels, aggregated across survey waves. As hypothesized, when aggregating data across survey waves, compared with England (52.5%), noticing any vaping campaigns or messages was more prevalent among youth in the United States (72.1%; AOR = 2.34, 95% CI = 2.24-2.44, *P* < 0.001) and Canada (65.3%; AOR = 1.62, 1.56-1.68, *P* < 0.001). Noticing was also greater in the United States than Canada (AOR = 1.45, 1.39-1.51, *P* < 0.001).

Considering the 17 individual channels, those most commonly selected were websites/social media, school and television/radio (Table I). Compared with England, noticing on most channels was more prevalent among youth in both the United States and Canada. However, noticing in leaflets/flyers, kiosks/temporary sales locations and bars/pubs was more prevalent in England than Canada. Comparing the United States and Canada, noticing on most channels was more prevalent in the United States, although noticing in taxis or buses/public transit was more prevalent in Canada.

#### Trends over time (Aim 2)

Figure 1 shows the trends in noticing any vaping campaigns/messages within each country, and Table II shows the associations in detail and in each of the 17 individual channels. As hypothesized, noticing any vaping campaigns increased between 2018 and February/March 2020 in each country (England: 48.0% to 56.5%, AOR = 1.09, 1.07-1.12, *P* < 0.001; Canada: 52.7% to 74.2%, AOR = 1.25, 1.22-1.28, *P* < 0.001; United States: 55.2% to 74.6%, AOR = 1.21, 1.18-1.24, *P* < 0.001) before decreasing (Canada) or plateauing (England, United States) between February/March 2020 and August 2022 (Fig. 1 and Table II).

**Table II.** Changes over time in the proportion of youth who reported noticing vaping campaigns or public health messages within England, Canada, and the United States

	England (*n* = 28 829)			Canada (*n* = 30 076)			United States (*n* = 33 434)		
	%(*n*)	AOR (95% CI)	*P*	%(*n*)	AOR (95% CI)	*P*	%(*n*)	AOR (95% CI)	*P*
Any noticing									
2018 (Aug-Sep)	48.0 (1881)	**REF**		52.7 (2044)	**REF**		55.2 (2260)	REF	
2019 (Aug-Sep)	49.3 (1776)	1.01 (0.99-1.04)	0.292	70.4 (2906)	**1.20 (1.17-1.23)**	**<0.001**	70.7 (2886)	**1.17 (1.14-1.20)**	**<0.001**
2020 (Feb-Mar)	56.5 (2446)	**1.09 (1.07-1.12)**	**<0.001**	74.2 (3176)	**1.25 (1.22-1.28)**	**<0.001**	74.6 (3945)	**1.21 (1.18-1.24)**	**<0.001**
2020 (Aug)	52.7 (2343)	**1.05 (1.02-1.08)**	**<0.001**	67.7 (2879)	**1.17 (1.14-1.19)**	**<0.001**	74.2 (4625)	**1.21 (1.18-1.24)**	**<0.001**
2021 (Feb-Mar)	53.0 (2368)	**1.05 (1.02-1.08)**	**<0.001**	64.5 (3033)	**1.13 (1.11-1.16)**	**<0.001**	74.6 (3939)	**1.21 (1.18-1.24)**	**<0.001**
2021 (Aug)	53.9 (2360)	**1.06 (1.03-1.08)**	**<0.001**	63.9 (2985)	**1.12 (1.10-1.15)**	**<0.001**	77.4 (3803)	**1.25 (1.22-1.28)**	**<0.001**
2022 (Aug)	53.2 (2346)	**1.06 (1.03-1.08)**	**<0.001**	63.0 (2796)	**1.11 (1.08-1.14)**	**<0.001**	74.1 (3125)	**1.21 (1.18-1.24)**	**<0.001**
On websites or social media, like Facebook, Twitter, YouTube, Instagram or Snapchat									
2018 (Aug-Sep)	22.0 (885)	**REF**		26.7 (1056)	**REF**		29.7 (1234)	**REF**	
2019 (Aug-Sep)	24.5 (920)	**1.03 (1.00-1.05)**	**0.020**	44.1 (1850)	**1.19 (1.16-1.22)**	**<0.001**	44.7 (1894)	**1.16 (1.13-1.19)**	**<0.001**
2020 (Feb-Mar)	29.8 (1310)	**1.09 (1.06-1.11)**	**<0.001**	48.6 (2102)	**1.24 (1.22-1.27)**	**<0.001**	48.9 (2686)	**1.21 (1.18-1.24)**	**<0.001**
2020 (Aug)	26.3 (1171)	**1.05 (1.02-1.07)**	**<0.001**	39.7 (1714)	**1.14 (1.11-1.16)**	**<0.001**	50.1 (3244)	**1.22 (1.20-1.25)**	**<0.001**
2021 (Feb-Mar)	27.3 (1233)	**1.05 (1.03-1.08)**	**<0.001**	39.2 (1855)	**1.13 (1.11-1.16)**	**<0.001**	53.1 (2800)	**1.26 (1.23-1.29)**	**<0.001**
2021 (Aug)	28.2 (1239)	**1.06 (1.04-1.09)**	**<0.001**	37.9 (1785)	**1.12 (1.09-1.14)**	**<0.001**	55.4 (2771)	**1.29 (1.26-1.33)**	**<0.001**
2022 (Aug)	29.2 (1312)	**1.08 (1.06-1.10)**	**<0.001**	37.9 (1678)	**1.12 (1.09-1.14)**	**<0.001**	52.0 (2282)	**1.25 (1.22-1.29)**	**<0.001**
At school									
2018 (Aug-Sep)	18.0 (656)	**REF**		28.9 (1097)	**REF**		28.3 (1182)	**REF**	
2019 (Aug-Sep)	20.6 (718)	**1.03 (1.01-1.05)**	**0.010**	44.3 (1782)	**1.17 (1.14-1.19)**	**<0.001**	37.2 (1496)	**1.10 (1.07-1.12)**	**<0.001**
2020 (Feb-Mar)	24.4 (1009)	**1.07 (1.05-1.09)**	**<0.001**	50.6 (2122)	**1.24 (1.21-1.27)**	**<0.001**	44.8 (2351)	**1.18 (1.15-1.21)**	**<0.001**
2020 (Aug)	24.7 (1053)	**1.07 (1.05-1.09)**	**<0.001**	43.8 (1862)	**1.16 (1.13-1.18)**	**<0.001**	44.5 (2775)	**1.17 (1.15-1.20)**	**<0.001**
2021 (Feb-Mar)	24.8 (1076)	**1.07 (1.05-1.09)**	**<0.001**	41.2 (1946)	**1.13 (1.11-1.16)**	**<0.001**	44.4 (2306)	**1.17 (1.14-1.20)**	**<0.001**
2021 (Aug)	24.9 (1078)	**1.07 (1.05-1.09)**	**<0.001**	40.8 (1893)	**1.13 (1.10-1.15)**	**<0.001**	47.1 (2214)	**1.21 (1.18-1.24)**	**<0.001**
2022 (Aug)	24.8 (1071)	**1.07 (1.05-1.10)**	**<0.001**	42.6 (1873)	**1.14 (1.12-1.17)**	**<0.001**	44.0 (1813)	**1.17 (1.14-1.21)**	**<0.001**
On television or radio									
2018 (Aug-Sep)	17.4 (708)	**REF**		19.9 (784)	**REF**		27.3 (1117)	**REF**	
2019 (Aug-Sep)	15.5 (566)	**0.98 (0.96-1.00)**	**0.046**	31.5 (1308)	**1.12 (1.07-1.15)**	**<0.001**	44 (1810)	**1.18 (1.06-1.21)**	**<0.001**
2020 (Feb-Mar)	20.9 (906)	**1.04 (1.02-1.06)**	**<0.001**	38.5 (1649)	**1.20 (1.09-1.23)**	**<0.001**	48 (2556)	**1.23 (1.09-1.26)**	**<0.001**
2020 (Aug)	18.0 (806)	1.01 (0.99-1.03)	0.480	29.7 (1271)	**1.10 (1.03-1.12)**	**<0.001**	43.3 (2772)	**1.17 (1.07-1.20)**	**<0.001**
2021 (Feb-Mar)	18.8 (846)	1.01 (0.99-1.03)	0.156	27.6 (1310)	**1.08 (1.04-1.10)**	**<0.001**	44.2 (2312)	**1.18 (1.11-1.21)**	**<0.001**
2021 (Aug)	16.3 (737)	0.99 (0.97-1.01)	0.201	26.8 (1268)	**1.07 (1.05-1.09)**	**<0.001**	46.5 (2271)	**1.21 (1.11-1.24)**	**<0.001**
2022 (Aug)	14.4 (654)	**0.97 (0.95-0.99)**	**0.001**	24.8 (1128)	**1.05 (1.04-1.07)**	**<0.001**	42.7 (1845)	**1.17 (1.08-1.20)**	**<0.001**
On billboards or posters									
2018 (Aug-Sep)	12.1 (470)	**REF**		15.7 (627)	**REF**		17.7 (724)	**REF**	
2019 (Aug-Sep)	13.0 (445)	1.01 (0.99-1.03)	0.279	23.4 (962)	**1.08 (1.06-1.10)**	**<0.001**	26 (1127)	**1.09 (1.06-1.11)**	**<0.001**
2020 (Feb-Mar)	16.3 (709)	**1.04 (1.03-1.06)**	**<0.001**	27.6 (1175)	**1.13 (1.10-1.15)**	**<0.001**	31.10 (1733)	**1.14 (1.12-1.17)**	**<0.001**
2020 (Aug)	15.2 (670)	**1.03 (1.01-1.05)**	**<0.001**	21.6 (916)	**1.06 (1.04-1.08)**	**<0.001**	30.8 (2015)	**1.14 (1.12-1.16)**	**<0.001**
2021 (Feb-Mar)	16.0 (715)	**1.04 (1.02-1.06)**	**<0.001**	20.9 (987)	**1.05 (1.04-1.07)**	**<0.001**	32.1 (1691)	**1.15 (1.13-1.18)**	**<0.001**
2021 (Aug)	15.7 (688)	**1.04 (1.02-1.05)**	**<0.001**	20.7 (958)	**1.05 (1.03-1.07)**	**<0.001**	33.7 (1712)	**1.17 (1.15-1.20)**	**<0.001**
2022 (Aug)	12.8 (576)	1.01 (0.99-1.02)	0.326	20.5 (930)	**1.05 (1.03-1.07)**	**<0.001**	31.9 (1346)	**1.16 (1.13-1.18)**	**<0.001**
At a chemist/pharmacy									
2018 (Aug-Sep)	14.8 (595)	**REF**		18.2 (729)	**REF**		16.2 (670)	**REF**	
2019 (Aug-Sep)	17.2 (630)	**1.02 (1.00-1.04)**	**0.014**	25.7 (1065)	**1.08 (1.06-1.10)**	**<0.001**	19.8 (890)	**1.04 (1.02-1.06)**	**<0.001**
2020 (Feb-Mar)	19.2 (861)	**1.05 (1.03-1.06)**	**<0.001**	28.9 (1277)	**1.11 (1.09-1.13)**	**<0.001**	21.1 (1264)	**1.05 (1.03-1.07)**	**<0.001**
2020 (Aug)	19.9 (897)	**1.05 (1.03-1.07)**	**<0.001**	22.3 (954)	**1.04 (1.02-1.06)**	**<0.001**	22.6 (1514)	**1.07 (1.05-1.09)**	**<0.001**
2021 (Feb-Mar)	20.4 (959)	**1.06 (1.04-1.08)**	**<0.001**	22.7 (1045)	**1.05 (1.03-1.07)**	**<0.001**	26.8 (1415)	**1.11 (1.09-1.13)**	**<0.001**
2021 (Aug)	21.1 (931)	**1.06 (1.04-1.08)**	**<0.001**	24.2 (1126)	**1.06 (1.04-1.08)**	**<0.001**	28.5 (1428)	**1.13 (1.11-1.16)**	**<0.001**
2022 (Aug)	16.4 (742)	1.02 (1.00-1.03)	0.074	22.8 (1008)	**1.05 (1.03-1.07)**	**<0.001**	23 (977)	**1.07 (1.05-1.10)**	**<0.001**
In shops/stores that sell e-cigarettes/vaping products									
2018 (Aug-Sep)	13.7 (496)	**REF**		**14.3 (588)**	**REF**		11.7 (487)	**REF**	
2019 (Aug-Sep)	13.4 (506)	1.00 (0.98-1.01)	0.667	**19.4 (836)**	**1.05 (1.03-1.07)**	**<0.001**	18.1 (823)	**1.06 (1.04-1.08)**	**<0.001**
2020 (Feb-Mar)	17.3 (774)	**1.04 (1.02-1.05)**	**<0.001**	20.1 (928)	**1.06 (1.04-1.08)**	**<0.001**	20.4 (1120)	**1.09 (1.07-1.11)**	**<0.001**
2020 (Aug)	18.4 (833)	**1.05 (1.03-1.07)**	**<0.001**	17.2 (739)	**1.03 (1.02-1.05)**	**<0.001**	18.9 (1257)	**1.07 (1.06-1.09)**	**<0.001**
2021 (Feb-Mar)	20.2 (932)	**1.07 (1.05-1.09)**	**<0.001**	17.8 (821)	**1.04 (1.02-1.06)**	**<0.001**	20.1 (1118)	**1.09 (1.07-1.11)**	**<0.001**
2021 (Aug)	19.4 (871)	**1.06 (1.04-1.08)**	**<0.001**	17.3 (812)	**1.03 (1.02-1.05)**	**<0.001**	21.3 (1120)	**1.10 (1.08-1.12)**	**<0.001**
2022 (Aug)	16.1 (726)	**1.02 (1.00-1.04)**	**0.013**	16.4 (733)	**1.02 (1.01-1.04)**	**0.010**	19.4 (865)	**1.08 (1.06-1.10)**	**<0.001**
In print newspapers or magazines									
2018 (Aug-Sep)	14.0 (545)	**REF**		13.7 (560)	**REF**		16.6 (665)	**REF**	
2019 (Aug-Sep)	13.4 (495)	0.99 (0.98-1.01)	0.452	20.9 (850)	**1.07 (1.06-1.09)**	**<0.001**	19.8 (880)	**1.03 (1.01-1.05)**	**0.003**
2020 (Feb-Mar)	16.4 (727)	**1.02 (1.01-1.04)**	**0.008**	22.4 (983)	**1.09 (1.07-1.11)**	**<0.001**	21.8 (1262)	**1.05 (1.03-1.07)**	**<0.001**
2020 (Aug)	14.2 (629)	1.00 (0.98-1.02)	0.819	15.4 (657)	**1.02 (1.00-1.04)**	**0.013**	19.0 (1223)	**1.02 (1.01-1.04)**	**0.013**
2021 (Feb-Mar)	14.1 (635)	1.00 (0.98-1.02)	0.983	14.8 (678)	1.01 (1.00-1.03)	0.065	20.7 (1089)	**1.04 (1.02-1.06)**	**<0.001**
2021 (Aug)	14.6 (626)	1.00 (0.99-1.02)	0.606	14.0 (655)	1.01 (0.99-1.02)	0.487	19.4 (1027)	**1.03 (1.01-1.05)**	**0.007**
2022 (Aug)	12.7 (579)	0.99 (0.97-1.00)	0.120	13.0 (588)	0.99 (0.98-1.01)	0.468	17.5 (745)	1.01 (0.99-1.03)	0.362
Outside shops/stores that sell e-cigarettes/vaping products									
2018 (Aug-Sep)	11.0 (407)	**REF**		12.5 (523)	**REF**		11.4 (457)	**REF**	
2019 (Aug-Sep)	11.2 (420)	1.00 (0.98-1.02)	0.903	19.3 (815)	**1.07 (1.05-1.09)**	**<0.001**	17.5 (821)	**1.06 (1.04-1.08)**	**<0.001**
2020 (Feb-Mar)	16.5 (737)	**1.06 (1.04-1.08)**	**<0.001**	19.2 (878)	**1.07 (1.05-1.09)**	**<0.001**	19.4 (1094)	**1.08 (1.06-1.10)**	**<0.001**
2020 (Aug)	16.9 (757)	**1.06 (1.04-1.08)**	**<0.001**	16.3 (695)	**1.04 (1.02-1.06)**	**<0.001**	18.2 (1227)	**1.07 (1.05-1.09)**	**<0.001**
2021 (Feb-Mar)	17.5 (810)	**1.07 (1.05-1.09)**	**<0.001**	16.5 (770)	**1.04 (1.03-1.06)**	**<0.001**	20.6 (1128)	**1.09 (1.07-1.12)**	**<0.001**
2021 (Aug)	18.6 (814)	**1.08 (1.06-1.10)**	**<0.001**	16.9 (773)	**1.05 (1.03-1.06)**	**<0.001**	20.8 (1133)	**1.10 (1.08-1.12)**	**<0.001**
2022 (Aug)	14.2 (664)	**1.03 (1.02-1.05)**	**<0.001**	15.8 (718)	**1.03 (1.02-1.05)**	**<0.001**	19.4 (874)	**1.08 (1.06-1.11)**	**<0.001**
In leaflets/flyers									
2018 (Aug-Sep)	13.7 (497)	**REF**		10.1 (635)	**REF**		11.7 (751)	**REF**	
2019 (Aug-Sep)	13.4 (532)	1.00 (0.98-1.01)	0.676	15.7 (756)	**1.06 (1.04-1.07)**	**<0.001**	16 (1108)	**1.04 (1.02-1.06)**	**<0.001**
2020 (Feb-Mar)	16.0 (497)	**1.02 (1.01-1.04)**	**0.009**	17.6 (517)	**1.08 (1.06-1.09)**	**<0.001**	18.9 (1175)	**1.07 (1.05-1.09)**	**<0.001**
2020 (Aug)	15.5 (713)	**1.02 (1.00-1.04)**	**0.039**	11.9 (572)	**1.02 (1.01-1.03)**	**0.003**	17.1 (1088)	**1.06 (1.04-1.07)**	**<0.001**
2021 (Feb-Mar)	16.3 (715)	**1.02 (1.01-1.04)**	**0.008**	12.1 (629)	**1.02 (1.01-1.04)**	**0.002**	20.1 (1111)	**1.08 (1.06-1.11)**	**<0.001**
2021 (Aug)	16.3 (730)	**1.02 (1.01-1.04)**	**0.008**	13.7 (567)	**1.04 (1.02-1.05)**	**<0.001**	20.5 (797)	**1.09 (1.07-1.11)**	**<0.001**
2022 (Aug)	13.5 (724)	1.00 (0.98-1.01)	0.722	12.5 (4090)	**1.02 (1.01-1.04)**	**0.001**	17.8 (6506)	**1.06 (1.04-1.08)**	**<0.001**
Taxis or buses/public transit									
2018 (Aug-Sep)	8.7 (341)	**REF**		11.5 (470)	**REF**		9.6 (407)	**REF**	
2019 (Aug-Sep)	9.2 (342)	1.00 (0.99-1.02)	0.556	17.9 (743)	**1.06 (1.05-1.08)**	**<0.001**	12.2 (570)	**1.03 (1.01-1.04)**	**0.004**
2020 (Feb-Mar)	13.3 (590)	**1.05 (1.03-1.06)**	**<0.001**	22.2 (949)	**1.11 (1.09-1.13)**	**<0.001**	14.5 (850)	**1.05 (1.03-1.07)**	**<0.001**
2020 (Aug)	11.5 (530)	**1.03 (1.01-1.04)**	**<0.001**	17.7 (757)	**1.06 (1.05-1.08)**	**<0.001**	14.2 (1006)	**1.05 (1.03-1.06)**	**<0.001**
2021 (Feb-Mar)	13.0 (602)	**1.04 (1.03-1.06)**	**<0.001**	16.0 (742)	**1.05 (1.03-1.06)**	**<0.001**	15.8 (884)	**1.06 (1.04-1.08)**	**<0.001**
2021 (Aug)	12.8 (580)	**1.04 (1.02-1.06)**	**<0.001**	16.6 (769)	**1.05 (1.04-1.07)**	**<0.001**	16.8 (926)	**1.07 (1.05-1.09)**	**<0.001**
2022 (Aug)	10.5 (482)	**1.02 (1.00-1.03)**	**0.019**	16.6 (728)	**1.05 (1.03-1.07)**	**<0.001**	14.8 (658)	**1.05 (1.03-1.07)**	**<0.001**
At kiosks or temporary sales locations (in shopping centres, parked in the street, other places, but not at specific events)									
2018 (Aug-Sep)	9.0 (336)	**REF**		8.6 (358)	**REF**		8.4 (345)	**REF**	
2019 (Aug-Sep)	9.9 (361)	1.01 (0.99-1.02)	0.252	13.6 (556)	**1.05 (1.03-1.06)**	**<0.001**	11.3 (529)	**1.03 (1.01-1.04)**	**0.001**
2020 (Feb-Mar)	12.9 (574)	**1.04 (1.02-1.06)**	**<0.001**	14.7 (640)	**1.06 (1.05-1.08)**	**<0.001**	8.4 (716)	**1.04 (1.02-1.05)**	**<0.001**
2020 (Aug)	12.3 (544)	**1.03 (1.02-1.05)**	**<0.001**	11.3 (481)	**1.03 (1.02-1.04)**	**<0.001**	11.3 (791)	**1.04 (1.02-1.05)**	**<0.001**
2021 (Feb-Mar)	14.3 (625)	**1.05 (1.04-1.07)**	**<0.001**	11.2 (496)	**1.03 (1.01-1.04)**	**<0.001**	12.3 (820)	**1.06 (1.05-1.08)**	**<0.001**
2021 (Aug)	13.3 (590)	**1.04 (1.03-1.06)**	**<0.001**	10.4 (468)	**1.02 (1.01-1.03)**	**0.004**	11.9 (766)	**1.07 (1.05-1.09)**	**<0.001**
2022 (Aug)	11.3 (516)	**1.02 (1.01-1.04)**	**0.004**	10.8 (474)	**1.02 (1.01-1.04)**	**0.002**	14.7 (572)	**1.04 (1.03-1.06)**	**<0.001**
At events like fairs, markets, festivals, sporting events or music concerts									
2018 (Aug-Sep)	6.6 (246)	**REF**		7.7 (336)	**REF**		8.5 (351)	**REF**	
2019 (Aug-Sep)	7.1 (262)	1.00 (0.99-1.02)	0.593	14.1 (575)	**1.06 (1.05-1.08)**	**<0.001**	12.7 (590)	**1.04 (1.02-1.06)**	**<0.001**
2020 (Feb-Mar)	9.6 (431)	**1.03 (1.02-1.04)**	**<0.001**	14.3 (637)	**1.07 (1.05-1.08)**	**<0.001**	14.3 (782)	**1.06 (1.04-1.07)**	**<0.001**
2020 (Aug)	8.1 (362)	**1.01 (1.00-1.03)**	**0.028**	10.4 (435)	**1.03 (1.02-1.04)**	**<0.001**	10.8 (744)	**1.02 (1.01-1.04)**	**0.003**
2021 (Feb-Mar)	9.0 (399)	**1.02 (1.01-1.04)**	**0.001**	8.5 (402)	**1.01 (1.00-1.02)**	**0.076**	13.8 (765)	**1.05 (1.03-1.07)**	**<0.001**
2021 (Aug)	9.9 (403)	**1.03 (1.02-1.05)**	**<0.001**	9.3 (436)	**1.02 (1.01-1.03)**	**0.003**	13.5 (748)	**1.05 (1.03-1.07)**	**<0.001**
2022 (Aug)	8.9 (412)	**1.02 (1.01-1.04)**	**0.001**	10.7 (491)	**1.03 (1.02-1.04)**	**<0.001**	14.5 (612)	**1.06 (1.04-1.08)**	**<0.001**
At the cinema/movies									
2018 (Aug-Sep)	4.9 (174)	**REF**		7.9 (329)	**REF**		9.8 (388)	**REF**	
2019 (Aug-Sep)	5.7 (214)	1.01 (1.00-1.02)	0.206	13.2 (538)	**1.05 (1.04-1.07)**	**<0.001**	13 (557)	**1.03 (1.01-1.05)**	**0.001**
2020 (Feb-Mar)	7.9 (330)	**1.03 (1.02-1.04)**	**<0.001**	14.2 (602)	**1.07 (1.05-1.08)**	**<0.001**	15.4 (779)	**1.06 (1.04-1.08)**	**<0.001**
2020 (Aug)	6.5 (282)	**1.02 (1.00-1.03)**	**0.009**	8.6 (361)	1.01 (1.00-1.02)	0.086	12.5 (823)	**1.03 (1.01-1.04)**	**0.001**
2021 (Feb-Mar)	7.3 (302)	**1.02 (1.01-1.04)**	**<0.001**	9.1 (394)	**1.02 (1.00-1.03)**	**0.012**	14.9 (762)	**1.05 (1.03-1.07)**	**<0.001**
2021 (Aug)	7.9 (355)	**1.03 (1.02-1.04)**	**<0.001**	9.9 (438)	**1.02 (1.01-1.04)**	**<0.001**	15.2 (774)	**1.06 (1.04-1.08)**	**<0.001**
2022 (Aug)	6.4 (293)	**1.01 (1.00-1.03)**	**0.015**	9.6 (408)	**1.02 (1.01-1.03)**	**0.003**	14.5 (594)	**1.05 (1.03-1.07)**	**<0.001**
In email or text messages									
2018 (Aug-Sep)	4.3 (156)	**REF**		7.3 (303)	**REF**		8.9 (355)	**REF**	
2019 (Aug-Sep)	4.7 (174)	1.00 (0.99-1.01)	0.547	11.9 (492)	**1.05 (1.03-1.06)**	**<0.001**	12.4 (569)	**1.03 (1.02-1.05)**	**<0.001**
2020 (Feb-Mar)	7.2 (315)	**1.03 (1.02-1.04)**	**<0.001**	11.2 (502)	**1.04 (1.03-1.05)**	**<0.001**	14.1 (772)	**1.05 (1.04-1.07)**	**<0.001**
2020 (Aug)	6.2 (275)	**1.02 (1.01-1.03)**	**0.001**	8.0 (330)	1.01 (1.00-1.02)	0.106	11.4 (739)	**1.03 (1.01-1.04)**	**0.001**
2021 (Feb-Mar)	7.4 (313)	**1.03 (1.02-1.04)**	**<0.001**	8.2 (367)	**1.01 (1.00-1.02)**	**0.045**	14.3 (740)	**1.05 (1.04-1.07)**	**<0.001**
2021 (Aug)	7.8 (328)	**1.04 (1.02-1.05)**	**<0.001**	8.5 (379)	**1.01 (1.00-1.03)**	**0.019**	14.2 (725)	**1.06 (1.04-1.07)**	**<0.001**
2022 (Aug)	6.8 (313)	**1.03 (1.01-1.04)**	**<0.001**	8.7 (376)	**1.02 (1.00-1.03)**	**0.014**	11.9 (528)	**1.03 (1.01-1.05)**	**0.001**
At work									
2018 (Aug-Sep)	4.6 (175)	**REF**		6.6 (277)	**REF**		6.9 (276)	**REF**	
2019 (Aug-Sep)	5.9 (221)	**1.01 (1.00-1.02)**	**0.039**	11 (440)	**1.04 (1.03-1.06)**	**<0.001**	9.4 (425)	**1.02 (1.01-1.04)**	**0.003**
2020 (Feb-Mar)	8.2 (365)	**1.04 (1.02-1.05)**	**<0.001**	12.3 (523)	**1.06 (1.04-1.07)**	**<0.001**	9.6 (542)	**1.03 (1.01-1.04)**	**<0.001**
2020 (Aug)	7.1 (322)	**1.03 (1.01-1.04)**	**<0.001**	7.9 (331)	**1.02 (1.00-1.03)**	**0.006**	8.0 (550)	1.01 (1.00-1.02)	0.139
2021 (Feb-Mar)	7.7 (353)	**1.03 (1.02-1.04)**	**<0.001**	8.7 (390)	**1.02 (1.01-1.04)**	**<0.001**	11.3 (607)	**1.04 (1.03-1.06)**	**<0.001**
2021 (Aug)	8.8 (389)	**1.04 (1.03-1.06)**	**<0.001**	8.8 (384)	**1.02 (1.01-1.04)**	**<0.001**	11.6 (611)	**1.05 (1.03-1.07)**	**<0.001**
2022 (Aug)	7.3 (354)	**1.03 (1.02-1.04)**	**<0.001**	9.4 (425)	**1.03 (1.02-1.04)**	**<0.001**	10.2 (442)	**1.03 (1.02-1.05)**	**<0.001**
In bars or pubs									
2018 (Aug-Sep)	6.6 (231)	**REF**		6.2 (281)	**REF**		6.1 (243)	**REF**	
2019 (Aug-Sep)	6.7 (266)	1.00 (0.99-1.01)	0.950	9.7 (391)	**1.03 (1.02-1.05)**	**<0.001**	7.9 (377)	**1.02 (1.00-1.03)**	**0.031**
2020 (Feb-Mar)	9.3 (410)	**1.03 (1.01-1.04)**	**<0.001**	10.7 (484)	**1.05 (1.03-1.06)**	**<0.001**	8.6 (484)	**1.02 (1.01-1.04)**	**0.001**
2020 (Aug)	9.5 (420)	**1.03 (1.02-1.04)**	**<0.001**	7.1 (287)	**1.01 (1.00-1.02)**	**0.047**	7.1 (494)	1.01 (1.00-1.02)	0.146
2021 (Feb-Mar)	9.2 (413)	**1.03 (1.01-1.04)**	**<0.001**	7.2 (316)	**1.01 (1.00-1.02)**	**0.034**	10.5 (557)	**1.04 (1.03-1.06)**	**<0.001**
2021 (Aug)	9.6 (425)	**1.03 (1.02-1.04)**	**<0.001**	7.4 (347)	**1.01 (1.00-1.03)**	**0.009**	9.6 (526)	**1.04 (1.02-1.05)**	**<0.001**
2022 (Aug)	8.3 (391)	**1.02 (1.00-1.03)**	**0.023**	7.9 (376)	**1.02 (1.01-1.03)**	**0.002**	8.2 (401)	**1.02 (1.00-1.03)**	**0.011**
In regular postal mail									
2018 (Aug-Sep)	4.2 (152)	**REF**		5.3 (225)	**REF**		6.7 (263)	**REF**	
2019 (Aug-Sep)	3.9 (159)	1 (0.99-1.01)	0.541	9.4 (375)	**1.04 (1.03-1.05)**	**<0.001**	8.6 (397)	**1.02 (1.00-1.03)**	**0.024**
2020 (Feb-Mar)	6.6 (291)	**1.02 (1.01-1.04)**	**<0.001**	8.5 (375)	**1.03 (1.02-1.05)**	**<0.001**	9.1 (511)	**1.02 (1.01-1.04)**	**0.001**
2020 (Aug)	5.6 (260)	**1.02 (1.00-1.03)**	**0.007**	5.8 (241)	1.01 (1.00-1.02)	0.078	7.3 (484)	1.01 (0.99-1.02)	0.377
2021 (Feb-Mar)	7.2 (303)	**1.03 (1.02-1.04)**	**<0.001**	6.3 (268)	**1.01 (1.00-1.02)**	**0.008**	10.1 (547)	**1.03 (1.02-1.05)**	**<0.001**
2021 (Aug)	7.7 (341)	**1.04 (1.02-1.05)**	**<0.001**	6.7 (293)	**1.02 (1.01-1.03)**	**0.001**	10.9 (533)	**1.04 (1.03-1.06)**	**<0.001**
2022 (Aug)	6.0 (293)	**1.02 (1.01-1.03)**	**0.001**	6.5 (291)	**1.01 (1.00-1.02)**	**0.009**	8.4 (370)	**1.02 (1.00-1.03)**	**0.040**

All data except n are weighted.

Estimates were obtained using Stata’s *margins* post-estimation command following a survey wave*country interaction term added to separate logistic regression models (one per outcome) adjusting for demographic covariates (age group, sex, race/ethnicity, perceived family financial situation, student status).

‘Other’ was not modelled as an outcome because it was reported by <1% of the sample.

a Wording differed according to country: At a [chemist (UK)/ pharmacy (CA, US)]; In [UK = leaflets, CA, US = flyers]; At the [UK = cinema/CA-US = movies].

Bolded values are where *P*<.05.

Trends were similar within each of the 17 individual channels (Table II). Noticing was most common on websites/social media (range 22–55%, depending on country and survey wave), at school (range 18–51%) and on television/radio (range 14–48%), again with the most pronounced increases observed in the United States and Canada between 2018 and February/March 2020.

Tables S3-S7 show the trends stratified by smoking/vaping. In the United States and Canada, trends among all subgroups were comparable to the full sample. In England, increases over time in noticing any education campaigns or messages were observed among those who had never smoked/vaped, ever but not past 30-day smoking/vaping, and past 30-day smoking/vaping only.

### Perceived valence of vaping campaigns in 2022: country differences and associations with harm perceptions (Aim 3)

**Fig. 2.**
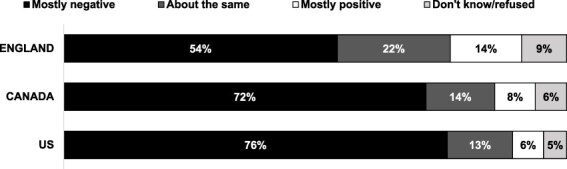
Proportion of youth within each country who perceived that vaping campaigns or public health messages were mostly negative, about the same or mostly positive among youth who noticed any vaping campaigns or public health messages in 2022 (*n* = 8267). Data are weighted.

Compared with England (54.4%), the perception that campaigns/messages were mostly negative towards vaping (versus otherwise) was more prevalent in the United States (76.3%; AOR = 2.78, 95% CI = 2.39-3.23, *P* < 0.001) and Canada (72.0%; AOR = 2.23, 1.95-2.55, *P* < 0.001, Fig. 2). Comparing the United States and Canada, the perception that campaigns/messages were mostly negative towards vaping was more prevalent in the United States (AOR = 1.25, 1.08-1.44, *P* = 0.003; Fig. 2). Table S8 shows the full adjusted logistic regression models and that similar country differences were observed when stratified by smoking/vaping subgroups.

Overall, less than half of youth accurately perceived vaping to be less harmful than smoking (41.6%). The accurate perception that vaping is less harmful than smoking was more prevalent among youth who perceived that campaigns/messages were mostly negative towards vaping (43.0%) compared with youth who did not perceive the campaigns/messages as mostly negative (41.4%) (AOR = 1.19, 1.05-1.33, *P* = 0.005); however, when stratified by smoking/vaping, this association was only significant among youth who had vaped but not smoked in the past 30 days (Table S9). When examining interactions, there was little evidence that the association between noticing negative education campaigns and accurate relative perceptions differed across countries (Canada versus United States difference: AOR = 0.87, 0.64-1.19, *P* = 0.389; England versus United States difference: AOR = 0.89, 0.65-1.21, *P* = 0.450) although accurate relative perceptions were more prevalent overall among youth in England (51.9%) than Canada (43.4%; AOR = 0.70, 0.61-0.79, *P* < 0.001) and the United States (34.8%; AOR = 0.48, 0.41-0.55, *P* < 0.001) (Table S9).

## Discussion

From 2018 through 2022, between half and three quarters of youth in England, Canada and the United States reported noticing education campaigns or public health messages about vaping. As hypothesized, noticing was most prevalent among youth in the United States, followed by Canada, and least prevalent among youth in England. Also as hypothesized, noticing increased between 2018 and February/March 2020 among youth in all three countries, but to a greater extent in the United States and Canada than in England. Youth mainly reported noticing vaping campaigns/messages on websites/social media, at school and on television/radio. Most campaigns/messages that were noticed were perceived to be mostly negative towards vaping in 2022, particularly in the United States.

Findings are consistent with the launch of well-funded national and regional youth vaping prevention campaigns on websites, social media, television and at schools in the United States and Canada [8–15, 18]. Noticing increased to the greatest extent in the United States, reaching 77% in August 2021, consistent with high expenditures. Findings are also consistent with widespread media coverage of the 2019 outbreak of lung injuries associated with vaping contaminated cannabis products [41–43] and with our previous work finding that misperceptions of the harms of vaping relative to smoking are pervasive among youth, and more pervasive in the United States and Canada than in England [6].

Vaping campaigns/messages that were noticed by youth were perceived to be mostly negative, consistent with the content of youth vaping prevention campaigns in the United States and Canada, as well as evidence reviews finding that youth-targeted campaigns aim to deter youth from trying vaping, often by highlighting the risks of vaping [1, 24, 25]. These findings may also reflect negativity bias, such that noticing and recalling negative information is easier than for neutral or positive information, particularly among youth [44]. Unexpectedly, and inconsistent with prior research [1, 24, 25], perceiving that messages portrayed vaping to be mostly negative was associated with accurate perceptions of vaping as less harmful than smoking, although this was only evident among youth who exclusively vaped, and there was little evidence for any association among youth who exclusively smoked, smoked and vaped or did neither. This study was cross-sectional and so the direction of associations cannot be established—it is possible that youth who vaped and believed vaping to be less harmful than smoking were more likely to remember negative campaigns because they conflicted with their existing beliefs and behaviours and stimulated feelings of dissonance or counter-arguing [45].

Certain channels may be more suitable for targeted communication of vaping information to youth, or to adults who smoke. Most youth in this study who noticed vaping campaigns/messages did so on websites/social media, at school and on television/radio. While information communicated via websites/social media and television/radio may also reach adults, schools are a useful venue for carefully-designed interventions, with accurate messages, to deter youth from vaping with minimal impact on adults who smoke, and evidence suggests that interventions in schools can change youth’s harm perceptions of vaping [1]. Conversely, campaigns/messages at work, in bars/pubs, and in the post/mail—which were noticed by few youth in this study—could be explored as potential channels for interventions that are intended to encourage adults who smoke to switch to vaping.

This study has limitations. First, the data collection period spanned the COVID-19 pandemic, which impacted behaviours including youth vaping [35], dominated public health messaging and disrupted education. Our hypothesis regarding trends over time was therefore restricted to the 2018 through February/March 2020 survey waves. Second, the outcome measure was noticing campaigns/messages in the past 12 months, which does not describe frequency or impact, has overlapping time periods for those survey waves that were 6 months apart and does not distinguish between education campaigns (which are strategic and active) and broader public health messages. Third, the high proportion of youth who reported noticing vaping campaigns/messages—particularly on websites/social media which was the leading channel—could be partially attributable to misreporting and/or conflation with noticing news stories or advertisements/marketing, which also increased over the study period [6, 46]. Fourth, samples were not probability based and survey weights differed between countries: data for Canada and the United States were weighted to reflect national smoking trends among youth, while data for England were not, due to lack of national smoking estimates among English youth aged 16–19; however, this would not have impacted within-country trends, and the large country differences that were observed in this study are unlikely to be an effect of survey weighting. Strengths of this study include the use of data from three countries with different expenditures on, and focuses of, vaping campaigns, and a large sample that allowed for subgroup analyses by smoking and vaping behaviours.

This study also has important implications. Vaping campaigns and public health messages were commonly noticed by youth in the United States, Canada and England, and most youth perceived vaping to be negatively portrayed. This suggests that campaigns aiming to deter youth from trying vaping are reaching their target audience. Most youth noticed on websites/social media and at school, and, as mentioned above, schools in particular could be a useful venue for carefully designed vaping campaigns specifically targeting youth. Building on our previous work examining trends in vaping perceptions since 2017 [6], this study found a further drop in the accurate perception that vaping is less harmful than smoking in 2022. Any campaigns or public health messages about vaping should therefore be accurate and balanced so as not to further exacerbate pervasive misperceptions of relative vaping harms.

## Supplementary Material

cyad044_Supp
